# The factors of adaptation to nursing homes in mainland China: a cross-sectional study

**DOI:** 10.1186/s12877-020-01916-x

**Published:** 2020-11-30

**Authors:** Changxian Sun, Yiting Yu, Xuxu Li, Yan Cui, Yaping Ding, Shuqin Zhu, Xianwen Li, Shen Chen, Rong Zhou

**Affiliations:** 1 Nanjing Medical University, Nanjing, China; 2 Jiangsu Vocational Institute of Commerce, Nanjing, China; 3 Landsea Lvy Elder Care Service, Nanjing, China

**Keywords:** Residential facilities, Adaptation, Resilience, Disease comorbidity, Social support

## Abstract

**Background:**

China is one of the most rapidly ageing countries and has the largest ageing population in the world. The demand for long-term care is increasing. Nursing home placement is one of the most stressful events in a person’s life. Although research on relocation adjustment has been conducted in many countries, few studies have been related to the predictors of nursing home adjustment in mainland China. This study aimed to identify the predictors of nursing home adjustment in the context of filial piety in mainland China.

**Methods:**

This was a descriptive study that employed a cross-sectional survey. A total of 303 residents from 22 nursing homes in Nanjing, China, were recruited. A structured questionnaire about residents’ characteristics, activities of daily living, social support, resilience, and nursing home adjustment was administered. Multiple linear regression was used to identify the predictors of adaptation to nursing homes.

**Results:**

The predictors of nursing home adjustment were the satisfaction with services(β = .158, *P* < .01), number of diseases(β = −.091, *P* < .05), length of stay(β = .088, P < .05), knowledge of the purpose of admission (β = .092, P < .05), resilience(β = .483, *P* < .001) and social support(β = .186, P < .001). The total explained variance for this model was 61.6%.

**Conclusion:**

Nursing staff members should assess the characteristics of residents to promote their better adjustment. Resilience had the most significant influence on the level of adaptation, which has been the primary focus of interventions to improve adjustment. The management of disease comorbidities in nursing homes should be standardized and supervised by the government. More volunteers from universities and communities should be encouraged to provide social support to residents. Moreover, a caring culture needs to be emphasized, and the value of filial piety should be advocated in nursing homes of East Asian countries.

**Supplementary Information:**

The online version contains supplementary material available at 10.1186/s12877-020-01916-x.

## Background

Population ageing is among the most critical global transformations. China is one of the fastest ageing countries and has the largest ageing population in the world. According to the latest report of the National Bureau of Statistics of China, older people aged over 60 accounted for 18.1% of the total population, and those over 65 represented 12.6% of the population by the end of 2019; this percentage is expected to rise to 26.9% of the total population by 2050 [1]. It was reported that there were nearly 30,000 aged care facilities and more than 7.46 million beds for aged care services in July 2019 [2]. The acceleration of population ageing will increase the pressure on social security and public services and continuously affect the social vitality, innovation dynamics, and potential economic growth rate, which is a significant risk and challenge to population development in the new era.

The demand for long-term care is increasing. Many older adults begin to lose the ability to take care of themselves owning to chronic disease, disability, and weakness [3]. Family care for older adults is reducing, with the development of the economy and the change of family structures [4]. Nursing homes are designed to provide a safe environment and continuous care. Relocation is considered to be one of the most stressful events that a person can experience, usually accompanied by depression, anxiety, loneliness, insomnia, and suicide attempts [5]. Transitions are complicated emotional regulation and are characterized by profound changes affecting one’s perception and understanding of the world and self [6]. Maladjustment to a new environment damages the quality of life and health of older people. The decision to move is a necessity rather than a choice [7]. People must accept the loss of familiar surroundings and adapt to the new environment. If nursing staff understand the variables affecting nursing home adjustment, they are more likely to help older adults achieve better adaptation, and interventions are more effective.

Nursing home adjustment is a process that changes over time [8]. Adapting to a new living environment requires an older adult to learn to follow new routines, participate in value activities, to establish and maintain new social relationships, and to manage personal property [9]. Xiao defined the operational concept of nursing home adjustment which included five dimensions: emotional distress, relationship development, acceptance of new residence, depressed mood, and feeling at home [10]. Nursing home adjustment varies depending on the length of stay, interventions should be taken during the most effective period because appropriate interventions are carried out promptly to improve effectively older adults’ quality of life in nursing homes [11]. Promoting factors of nursing home adjustment included satisfaction with the facility and the care that they received. The greater the satisfaction the older people living in a nursing home had, the better their adaptation [12]. Unplanned admissions to nursing homes usually caused severe health outcomes for older people, such as anxiety, emotional distress, and increased confusion [13]. It was reported that older adults with planned nursing home admissions progressed faster in the adjustment phase than older adults with unplanned admissions [8]. The physical health of older people in nursing homes was related to mental health problems, causing maladaptation [14]. This adaptation has been reported to be associated with a decline in physical and cognitive function [15]. In the case of involuntary relocation, the adjustment to long-term care facilities became very hard and there was a trend towards increased mortality [14, 16]. Over the past few decades, a few studies have been conducted to determine the factors that influence the adaptation of older adults. In South Korea, factors influencing adaptation included self-efficacy, the quality of the facility, depression, the decision to move, perceived health status, age, self-reported health, preconceptions about nursing homes, emotional support from staff and other residents, and general satisfaction with the facility [14, 17]. In a synthesis study, the facilitators and inhibitors of adjustment covered four themes: resilience of the older person, interpersonal connections and relationships, a feeling that “this is my new home”, and a view of the care facility as an organization [18]. However, Fitzpatrick pointed out that the heterogeneity of research limited the generalizability of the findings, such as the use of multiplicities of terms and concepts, quality of different methods, timelines for different investigation of the transitions [18]. Transition to long-term care facilities causes variations in their responses to relocation since older adults have different socioeconomic statuses, educational levels, careers, and cultural backgrounds [19]. Sociocultural values and ethnic background were also predictors of adjustment [20]. In the context of filial piety, older Chinese adults expected their children to take care of them at home, however, relocation caused the ambivalence of social expectations among Chinese families; this traditional virtue had a profound impact on nursing home adjustment [21]. Moreover, although the factors identified in previous studies affecting adaptation were similar, there were differences in the most influential factors due to different regions and ethnicities [14].

Research from Taiwan, China, showed the most important factors affecting overall adaptation were sufficient funding for admission, voluntary admission, and the number of roommates. However, the local culture, welfare systems, and policies in Taiwan are different from those in mainland China. So far, there has been few studies on nursing home adjustment in mainland China. The purpose of this study was to identify the predictors of older adults’ adjustment so that nursing staff could promote a healthy transition. The findings may also be used to inform Chinese geriatric care policy to help older Chinese people and their families adapt more easily to the new home environment.

## Methods

**Fig. 1:**
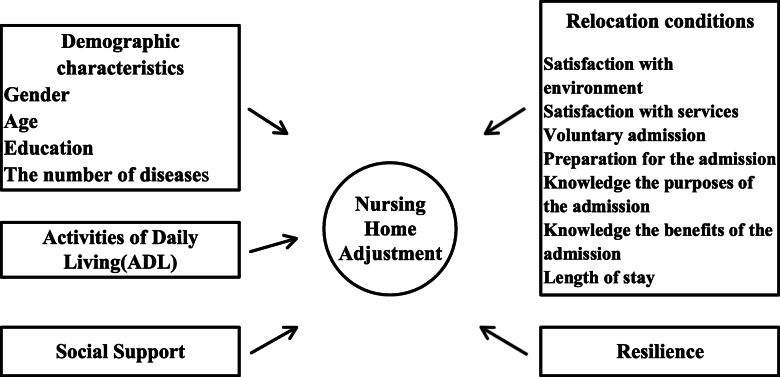
Research Framework

A cross-sectional descriptive correlation study was conducted to investigate the adaptive status of older people in nursing homes and its influencing factors. Figure 1 showed the framework of the research. The factors examined in this study were selected based on the results of previous studies. The following factors on nursing home adjustment was investigated: demographic characteristics (sex, age, education, and the number of diseases), relocation conditions (satisfaction with environment, satisfaction with services, voluntary admission, preparation for admission, knowledge of the purpose of admission, knowledge of the benefits of admission, and length of stay), activities of daily living (ADL), resilience, and social support.

### Ethical considerations

Ethical approval was obtained from the Ethics Committee of Nanjing Medical University (No. 2018(650)). Participants were informed about the purpose and methods of the study. Their participation was voluntary, and they had the right to withdraw from the study at any time. Written informed consent would be obtained from all participants and their family members. Some residents were illiteracy, who still could read and write but did not get informal education from school.

### Settings and samples

A convenient sampling method was used in this study (Jager, 2017) [22]. The participants were recruited from 22 nursing homes in Nanjing, Jiangsu Province, China. These nursing homes are located in urban, suburban, and rural areas. The criteria for inclusion in the study were age > 60 years, no cognitive impairment, and full capability to communicate verbally. Older people with mental retardation were excluded. The sample size should be 10–20 times the number of variables when using multivariable linear regression (Hu 2017) [23]. One way to carry out a multivariable regression is to include all potentially relevant independent variables in the model (complete model). The problem with this method is that the number of observations that can practically be made is often less than the model requires. In general, the number of observations should be at least 20 times greater than the number of variables under study [24]. Fourteen variables were studied, so the necessary sample size was estimated to be 280. A total of 303 residents met the criteria and were recruited as participants.

### Data collection

Twelve students majoring in service and management for older adults underwent standardized training and completed a 3-day presurvey before the commencement of the study to ensure that every investigator was qualified. Data were collected from June to September 2019. The questionnaires were administered in one-on-one interviews by trained interviewers because participants had limited literacy and vision.

### Measurement

A structured questionnaire comprising five parts was used to collect data. The structured questionnaire included the following five subsections: demographic questionnaire, Barthel Index, Social Support Rating Scale, Resilience Scale, and Nursing home adjustment.

1. Demographic questionnaire: this questionnaire was used to collect demographic information regarding age, sex, educational background, the number of diseases, length of stay, and some relocation conditions
2. The Chinese version of the Barthel Index (BI) [25]: The BI is used to measure the functional status of the activities of daily living (ADL), which consist of feeding, dressing, bathing, grooming, toileting, bowel control, bladder control, transfer between the wheelchair and bed, ambulating, and using stairs. Scores range from 0 to 100. A higher score indicates a higher level of resident independence. The scores are divided into five groups: < 20, indicating totally dependent; 20–39, indicating very dependent; 40–59, indicating partially dependent; 60–79, indicating minimally dependent and 80–100, indicating independent. The Cronbach’s α of the scale is 0.916. The split-half reliability is 0.8373.
3. Social Support Rating Scale (SSRS):(Xiao,1994) [26]: The SSRS is the most commonly used scale to examine the extent to which individuals receive and use support in their social lives. There are ten items and three dimensions, including objective support (3 items), subjective support (4 items), and utilization of social support (3 items). The total score is the sum of the 10 items. Each item is rated from low to high on a 4-point scale, with an overall score of 40. The higher the score, the higher the social support one receives. The Cronbach’s α of the scale is 0.81. The test–retest reliability is 0.92. The correlation coefficient between the SSRS and SCL-90(Symptom Checklist 90) scores is-0.1848.
4. The Chinese version of the Resilience Scale (RS-14): (Ni, 2013) [27] This scale is the most widely used scale to measure resilience. The scale has two dimensions, including personal competence (10 items) and acceptance of self and life (4 items). It uses a 7-point Likert scale ranging from 1 (strongly disagree) to 7 (strongly agree). The total score is the sum of the 14 items and ranges from 14 to 98. The higher the score is, the better the level of resilience. The Cronbach’s α of the scale is 0.93. The split-half reliability and test–retest reliability of the RS-14 are both 0.82. The correlation coefficient between the RS-14 and SF-36 scores is 0.82.
5. The Chinese version of the Nursing Home Adjustment Scale (NHAS) [10]: This scale is a reliable and useful tool to evaluate the level of nursing home adjustment in China. The NHAS has twenty-three items and five dimensions, including emotional distress (2 items), relationship development (7 items), acceptance of new residence (6 items), depressed mood (6 items), and feeling at home (2 items). It uses a 5-point Likert scale (from strongly disagree =1 to strongly agree =5), with higher scores indicating better adaptation. The Cronbach’s alpha of the Chinese version of the NHAS is 0.87. The ICC of the test–retest measure is 0.72 for the total scale.

### Data analysis

Data were analyzed using SPSS version 23.0. Descriptive analysis was used to describe the general characteristics. Multiple linear regression [28] was conducted to explore the influencing factors of adjustment for older people living in nursing homes. Educational background was entered as a dummy variable.

## Result

The demographic characteristics of the participants are shown in Table 1. There were more female subjects (61.1%) than male subjects (38.9%). The mean age of the participants was 82.4 (SD = 8.1) years old. Regarding the level of education, 43% of the elderly people had only an elementary school education or less. Of the participants, 72.3% were admitted voluntarily, 63.7% prepared for admission, 87.1% knew the purpose of admission, 75.6% knew the benefit of living in the nursing home, and over 95% were satisfied with the environment and service of the nursing home. The median length of stay in the nursing home was nine months, and the length of stay ranged from 0.1 to 96 months. The mean number of diseases was 1.67 ± 1.12, and 47.4% of participants had disease comorbidities in nursing homes.

**Table 1:** Distribution of characteristics of participants (*N* = 303)

Characteristics category	N(%)	Median	Mean ± SD
Sex			
Male	118 (38.9)		
Female	185 (61.1)		
Age (year)			82.4 ± 8.1
60–69	22 (7.3)		
70–79	76 (25.1)		
80–89	144 (47.5)		
> 90	61 (20.1)		
Education			
Illiteracy	65 (21.5)		
Elementary school	65 (21.5)		
Middle school	37 (12.2)		
High school	68 (22.4)		
College and above	68 (22.4)		
Satisfaction with environment			
NO	10 (3.3)		
YES	293 (96.7)		
Satisfaction with services			
NO	14 (4.6)		
YES	289 (95.4)		
Voluntary admission			
NO	84 (27.7)		
YES	219 (72.3)		
Preparation for the admission			
NO	110 (36.3)		
YES	193 (63.7)		
Knowledge of the purpose of admission			
NO	39 (12.9)		
YES	264 (87.1)		
Knowledge of the benefits of admission			
NO	74 (24.4)		
YES	229 (75.6)		
Length of stay (months)		9 (4–16)	
Number of diseases			1.67 ± 1.12
ADL (BI)			72.4 ± 26.6
<20(Totally dependent)	12 (4.0)		
20–39(Very dependent)	31 (10.2)		
40–59(Partially dependent)	40 (13.2)		
60–79(Minimally dependent)	65 (21.5)		
80–100(Independent)	155 (51.1)		
RS			60.0 ± 15.7
SSRS			33.5 ± 8.1
NHAS			80.5 ± 17.4

*ADL* Activities of Daily Living, *BI* Barthel Index, *RS* Resilience Scale, *SSRS* Social Support Rate Scale, *NHAS* Nursing Home Adjustment

The levels of ADL, social support, resilience, and NHAS are shown in Table 1. The mean ADL score (Barthel Index) was 72.4 (SD = 26.6), and the participants demonstrated a mild level of dependency. The mean RS score was 60.0 (SD = 15.7). The mean SSRS score was 33.5 (SD = 8.1), suggesting that participants were given moderate levels of social support. The average NHAS score was 80.5 (SD = 17.4), with a mean emotional distress score of 7.2 (SD = 2.2), a mean relationship development score of 23.7 (SD = 6.1), a mean acceptance of new residence score of 22.0 (SD = 4.7), a mean depressed mood score of 20.5 (SD = 6.2), and a mean feeling at home score of 7.1 (SD = 2.0).

The correlations between the participants’ adaptation to nursing homes and related factors are shown in Table 2. All 14 independent variables, as a group, were entered into multiple regression models. There was no collinearity among the independent variables. The statistically significant predictors of nursing home adjustment were satisfaction with services (β = .158, *P* < .01), number of diseases (β = −.091, *P* < .05), length of stay (β = .088, P < .05), knowledge of the purpose of admission (β = .092, P < .05), RS score (β = .483, *P* < .001), and SSRS score (β = .186, P < .001). A statistically significant regression equation was revealed (F = 29.523, *P* < 0.001), which explained 61.6% of the variance in nursing home adjustment.

**Table 2:** Multiple regression analysis of predictors of adaptation to nursing home life (N = 303)

	Unstandardized Coefficients		Std. Coefficients	t	Sig.	Collinearity Statistics		Adj. R2	F/p
	B	Std. Error	β			Tolerance	VIF		
Constant	−1.656	8.725		−.0190	0.850			0.616	29.523 P < 0.001
Education									
Elementary school	−2.243	1.964	−0.053	−1.142	0.254	0.589	1.697		
Middle school	−.298	2.333	−0.006	−0.128	0.898	0.657	1.523		
High school	−1.685	2.012	−0.040	− 0.837	0.403	0.543	1.84		
College and above	−0.580	2.081	−0.014	−0.279	0.781	0.508	1.968		
Satisfaction with environment	5.548	4.680	0.057	1.185	0.237	0.548	1.825		
Satisfaction with services	13.034	4.194	0.158	3.108	0.002	0.494	2.024		
Age	0.124	0.083	0.058	1.493	0.136	0.855	1.169		
Sex	1.060	1.351	0.030	0.784	0.434	0.883	1.133		
Number of diseases	−1.398	0.589	−0.091	−2.373	0.018	0.874	1.145		
Length of stay (months)	0.108	0.047	0.088	2.326	0.021	0.896	1.116		
Voluntary admission	2.800	1.613	0.072	1.735	0.084	0.734	1.362		
Preparation for the admission	1.797	1.581	0.050	1.136	0.257	0.662	1.510		
Knowledge of the purpose of admission	4.749	2.119	0.092	2.241	0.026^*^	0.760	1.315		
Knowledge of the benefits of admission	1.577	1.676	0.041	0.941	.348	0.684	1.461		
ADL (BI)	0.006	0.025	0.009	0.222	0.825	0.856	1.168		
RS	0.533	0.053	0.483	10.149	< 0.001	0.562	1.780		
SSRS	0.401	0.101	0.186	3.989	< 0.001	0.583	1.715		

*ADL* Activities of Daily Living, *BI* Barthel Index, *RS* Resilience Scale, *SSRS* Social Support Rate Scale

## Discussion

This study sought to explore the predictors of adaptation to nursing homes in mainland China. The findings provide a useful understanding of residents’ adaptation to a nursing home. We identified the main predictors to be satisfaction with institutional service, the number of diseases, length of stay, knowledge of the purpose of admission, resilience, and social support. Among those factors, the most influential was resilience. According to previous studies, satisfaction, health status, length of stay, resilience, and support were generally similar predictors [14, 17, 18, 29]. These results confirmed available evidence showing that disease comorbidities and knowledge of the purpose of admission are the critical points of interventions. These findings further support that resilience, a core variable, served as the underpinning for strategies to promote adaptation [30].

In this study, 95.4% of participants were satisfied with institutional services, which was also a significant predictor of adjustment. The greater the residents’ perceived satisfaction with the facility was, the better their adjustment to nursing home life, which was similar to the findings of Lee [31]. The “Basic specification of service quality for senior care organization” by the Chinese government proposed the direction of the development of senior care be focused on standardization and specialization; the service level of employees; “safe, honest and high-quality” service; and the encouragement of building service brands [32]. With Chinese mainstream culture deeply rooted in Confucian ideology, filial piety is a core virtue of the society [33]. Thus, most facilities advocate creating a caring culture of filial piety and an atmosphere of home, helping residents gain a sense of belonging. Fitzpatrick also reported that it was warranted for nursing staff to understand how organizational culture influences transition and to create a caring culture for older people [18].

This study showed that the more diseases older adults had, the lower their level of adaptation to nursing homes. This result indicated that 47.4% of participants in nursing homes had disease comorbidities. The most prevalent comorbid mental and behavioural conditions in the Australian aged care population were dementia and depression, which presented a challenge for the management of multiple chronic conditions [34]. The prevalence of comorbidity reaches 60% in people aged over 65 in China, which significantly reduces the health of older adults, increases readmission rates and the potential socioeconomic burden, and even increases the risk of death [35]. The older people in nursing homes in Beijing have higher cognitive levels of chronic diseases than those living at home; however, some facilities in undeveloped areas just provide simple services with low quality and lack the ability to manage chronic illness [36]. The concept of health should be integrated into the service for older people through the new ‘Combination of medical service and care’ policy [37]. This policy has promoted collaboration between nearby hospitals and nursing homes, and brought numerous benefits to residents in cities. Residents in some nursing homes can obtain medical services, such as appointments with doctors, rehabilitation, Chinese medicine treatment, and drug service in some nursing homes. Moreover, national medical insurance can also be used in nursing homes, avoiding residents’ being rushed to large hospitals. However, the management of disease comorbidities should be further enhanced in nursing homes, which would allow residents to gradually perceive the benefits of nursing homes and achieve better adjustment.

Residents’ life adjustments might vary based on the length of stay and occur over time. This study indicated that a longer length of stay was a facilitator of transition. A longer length of stay might lead to better life adjustment for residents, but life adjustment could be an on-going dynamic process rather than a static state after stabilization [11]. A longer length of stay was associated with better communication with staff as well as residents’ feelings of being respected in Singapore [38]. Residents who stayed for twelve months or more showed the highest NHAS scores [11]. Brooke reported that the disorganization phase lasted 6–8 weeks after relocation [39]. Thus, interventions are given to older adults who have lived for less than one year to promote adaptation, especially in the initial phase of transition (e.g., within two months) [11, 29].

Twelve percent of the participants did not know the purpose of the move, causing lower adjustment. On the one hand, some residents did not perceive their functional status to have changed and desired to stay at home. On the other hand, their family members did not inform them or consult with them and made decisions directly. Older adults felt abandoned and upset, showed resistance to the new environment, and reported dissatisfaction with the facilities. This suggested that family members and nursing staff should help residents understand the true purpose of their admission, which is also the first step of promoting adaptation. We should patiently wait for older adults’ perceptions to change and learn to be respectful of their minds, thereby adhering to the culture of filial piety.

This study suggested that resilience was the primary focus of interventions and significantly improved nursing home adjustment. Resilience refers to a person’s ability to successfully adapt to challenges in life that are often described as negative, traumatic, or stressful [40]. Traits found to be plentiful in resilient people are independence, above average intelligence, positive attitude, optimism, a strong sense of self, varied interests, and involvement in many social activities [41]. Personal resilience may play a role in individual’s insight and ability to develop these facilitative strategies. Resilience can be learned. The nursing staff may guide residents to reflect on their capabilities, agree with the need for the move and have an attitude of hope [30]. Most of the participants were willing to share reasons for moving and their concerns about being a burden on their family. The development of an intervention that targets resilience promotes older people’s well-being. Older adults tend to talk about their life stories to summarize their lives. The process of constructing and reinterpreting past events in light of recent events is critical to developing resilience. Mager conducted five structured weekly storytelling groups with eight older adults and reported improvement in resilience and happiness [41].

The more the residents perceived support, the better they adjusted to nursing home life, which was consistent with a previous study [17]. Social support is described as various kinds of help or assistance from family, friends, and others. Nursing home admission caused a feeling of rejection and severe loneliness owing to separation from family [13]. Ties to family and friends influence the adjustment [42]. Family connections can maintain such relationships to facilitate older adults’ adaptation to nursing homes. However, it is not very easy for many older adults to establish new social networks. Because residents around are too old with cognitive impairment, and staff members are busy with basic care. Older adults receive limited emotional support from staff and other residents, which creates a desire to return to their previous home situation [5]. Social support, positive social interaction with others, and supportive social policies could give rise to resilience [43]. In this study, social support was the second vital focus for intervention. In China, university students regularly visit residents voluntarily, talk with them, and perform group activities, providing emotional support and companionship. Young adults interact with older adults with enthusiasm and provide a certain degree of social support, practising filial piety. Currently, an increasing number of social workers in nursing homes are evaluating and tracking residents’ physical and psychosocial changes dynamically within 15–30 days after relocation and then making tailored care plans to assist the transition as routine work.

There were some limitations to this study. The generalizability of the findings was limited because the data were collected through the convenience sample sampling. Participants should be recruited from different regions of the country to confirm the generalizability in future studies. The use of a cross-sectional survey limited the exploration of residents’ adaptation over time. A longitudinal study is needed to track changes in adjustment levels and factors influencing the adjustment over time. In addition, the number of diseases was underestimated because of incomplete health records in nursing homes.

## Conclusion

Satisfaction with services, number of diseases, length of stay, knowledge of the purpose of admission, resilience, and social support were significant predictors of nursing home adjustment. Nursing staff members should assess the characteristics of residents to promote their better adjustment. Resilience had the most significant influence on the level of adaptation. Nursing staff designed appropriate programmes to enhance resilience and improve adjustment, such as storytelling. The management of disease comorbidities in nursing homes should be standardized and supervised by the government. Knowledge of the true purpose of admission is the first step to promote adaptation. A caring culture needs to be emphasized, and the value of filial piety should be advocated in nursing homes of East Asian countries. A caring culture needs to be emphasized, and the value of filial piety might be advocated in nursing homes of East Asian countries. In the future, a longitudinal study is needed to explore the trajectory of transition, and interventions fostering adaptation, such as intergenerational support programs by college students, should be developed.

## Supplementary Information

**Additional file 1.** Demographic Questionnaire.

## Data Availability

Our data may not be shared directly, because it is our teamwork; informed consent should be attained from all the team members. Our data or material may be available after contacting the corresponding author or first author.

## Abbreviations

ADL: Activities of Daily Living
BI: Barthel Index
RS: Resilience Scale
SSRS: Social Support Rate Scale
NHAS: Nursing Home Adjustment
