# Supplementary material for: The factors of adaptation to nursing homes in mainland China: a cross-sectional study
Source: BMC Geriatr. 2020 Nov 30;20:517. doi: 10.1186/s12877-020-01916-x (PMC7708906; doi:10.1186/s12877-020-01916-x)
Supplement: Supplementary file 1 — Additional file 1. Demographic Questionnaire. [file 12877_2020_1916_MOESM1_ESM.docx]

1. Demographic questionnaire

The questionnaire was developed for this study and used to collect demographic information.

| Age | year |
| --- | --- |
| Gender | 1 □Male 2、□Female |
| Education | 1、□Illiteracy 2、□Elementary school 3、□Middle school  4、□High school 5、□College and above |
| Number of diseases |  |
| Length of stay | months |
| Voluntary admission | 1、□Yes 2、□No |
| Preparation for the admission | 1、□Yes 2、□No |
| Knowledge of the purpose of admission | 1、□Yes 2、□No |
| Knowledge of the benefits of admission | 1、□Yes 2、□No |
| Satisfaction with environment | 1、□Yes 2、□No |
| Satisfaction with services | 1、□Yes 2、□No |
